# An exploratory study of the views of Ugandan women and health practitioners on the use of sonography to establish fetal sex

**DOI:** 10.4314/pamj.v9i1.71214

**Published:** 2011-08-01

**Authors:** Aloysius Gonzaga Mubuuke

**Affiliations:** 1 Radiology Department, School of Medicine, College of Health Sciences, Makerere University, Kampala, Uganda

**Keywords:** Fetal sex, sonography, Uganda

## Abstract

**Introduction:**

Ultrasound is now part of routine care for pregnant women in Uganda, and is one of a range of techniques used in screening during pregnancy. However, it differs from most others screening procedures because it allows women to view their babies. Unfortunately, the recipients of this technology are seldom asked about it. This study aimed at finding out the knowledge, attitudes and practices of pregnant women towards prenatal sonography.

**Methods:**

The study was exploratory and descriptive, using interviewer-administered questionnaires. Thematic analysis was employed.

**Results:**

The health professionals interviewed discouraged the idea of disclosing fetal sex unless it is justifiably indicated for medical reasons. However, the women in this study supported the idea of being told the sex of the baby in order to plan for the necessary items they need.

**Conclusion:**

There is need for a policy to be made not to disclose fetal sex to parents as this raises numerous ethical concerns. Health workers, women and the general public need to be sensitized about the dangers of this practice as well.

## Introduction

There has been increased medicalization of pregnancy globally due to advances in technology in the field of healthcare and most especially in obstetric care [1]. Routine obstetric ultrasound has been one of the most important advances in antenatal care worldwide [2–5]. However, it has been reported that innovative medical technologies like obstetric sonography have the potential to raise social, ethical and economic dilemmas for both health workers and the recipients of health services [4].

There has been increased medicalization of pregnancy globally due to advances in technology in the field of healthcare and most especially in obstetric care [1]. Routine obstetric ultrasound has been one of the most important advances in antenatal care worldwide [2–5]. However, it has been reported that innovative medical technologies like obstetric sonography have the potential to raise social, ethical and economic dilemmas for both health workers and the recipients of health services [4].

In Uganda, health care delivery has been decentralized from the top to the bottom with the aim of bringing services nearer to the people having national referral hospitals at the top and low-level health centres up to villages [6]. In most of these health care facilities including private health care facilities, routine obstetric sonography has been fully embraced. As a result of this most women willingly go for obstetric scans sometimes even without the doctor‘s advice. Whynes further expounds on this that, most women now accept the scan uncritically because of the enormous expectations they have, but most especially viewing their babies live on the screen and knowing the fetal sex [7].

Although fetal sex can be determined as early as 13 to 14 weeks, most experts agree that the sonographic detection rate sharply increases after 18 weeks of gestation [8]. Some reasons of detecting fetal gender are abnormal genitalia in X-linked disorders, testicular feminization, pseudo-hermaphroditism and hydrocele [8–15]. Fetal sex can be detected sonographically and the genitalia can be predicted successfully 83.5% of the time between 16-20 weeks gestation [16].

Determination of fetal sex is not only done for parental curiosity but also has many medical advantages. Accurately assessing fetal sex can assist in assigning zygosity in twin pregnancies. Ambiguity of the genitalia can occasionally be detected sonographically after detecting other abnormalities, because of a relevant family history. Some cases are diagnosed after careful evaluation of fetal gender because of an antenatal discrepancy between the fetal karyotype and the genital anatomy. In women at risk of X-linked genetic disease or ambiguous development of the external genitalia early gender assignment may give parents the option to avoid invasive testing in up to half of cases where the fetus would not be affected [14, 15].

The moral issues concern whether it is “right” to be determining the sex of a fetus before it is born, and if that knowledge also determines whether or not the baby lives. Using prenatal diagnosis generates information which society has not yet learned to deal with. The issue of who is more at “fault” is also raised. If a fetus is aborted because of its sex, does the responsibility lie more in the hands of the doctor who provided the sex information, or by the doctor who performs the abortion?[15]. There are social issues that are raised by sex determination tests. In some cultures, the higher status of males goes beyond the male/female salary inconsistencies. Although the approval rate for sex selection (using technologies for sex determination to abort the fetus) is low in the U.S.A, Canada, and The Western countries. Family size is decreasing and there is still a preference for firstborn sons. Boss also states that it can be detrimental to one's social status to be female in some countries [17].

However, revealing fetal sex to the mothers may prove detrimental and there might be stronger after effects than anticipated. Therefore, the ethical issues surrounding this subject need to be documented. The purpose of this study was to establish the ethical implications of using sonography to reveal fetal gender to the mothers. Throughout this study, the words “sonography”, “scan” and “ultrasound” may be used interchangeably.

## Methods

### Site

The study was conducted at Mulago Hospital in Uganda.

### Design

It was a cross-sectional exploratory study involving two groups of participants; professionals who do ultrasound scanning who include Radiologists and Sonographers and women who receive the ultrasound service. All participants who consented were included in the study. Semi-structured key-informant individual interviews were conducted. Individual interviews help to collect insightful descriptions from participants [18, 19]The questions asked explored issues like the advantages and disadvantages of revealing fetal gender at ultrasound, ethical and social issues surrounding this and the way forward. Each interview began with obtaining consent from the participant, explanation of the study followed by a discussion of any concerns. Participants were assured of the confidentiality of their opinions. The tape-recorded interview followed the completion of the aforementioned tasks. Demographic information for each participant was also collected. Open-ended questions were used and responses tape-recorded. The questions asked explored issues like the advantages and disadvantages of revealing fetal gender at ultrasound, ethical and social issues surrounding this and the way forward. Each interview began with obtaining consent from the participant, explanation of the study followed by a discussion of any concerns. Participants were assured of the confidentiality of their opinions. The tape-recorded interview followed the completion of the aforementioned tasks. Demographic information for each participant was also collected

### Sample size

Consecutive sampling was used. Thirty (30) Sonography professionals and fifty (50) women were included in the study. By the time of reaching at this number of participants, data saturation had been reached and all responses had become repetitive with no new themes coming up.

### Data analysis

Thematic content analysis was used. This involved content analysis to extract the meanings of the informants, and also transcription. Raw data was proof-read against audio-taped interviews and coded into categories of similar meaning. This technique is one Wilson collectively refers to as analytic description [20]. Categories were established, resulting into content themes, consistent with the value of thematic content analysis in qualitative methods [21, 22]. These themes summarized the meaning of the data which addressed the aim of the study.

### Ethical issues

Permission to carry out this study was obtained from the Radiology department research committee.

## Results

Opinions got from the sonography professionals (Radiologists and Sonographers) and the women who do the scans varied widely. While all sonography professionals discouraged the idea of telling mothers the sex of the fetus, all the women interviewed expressed strong desire to know the fetal sex at ultrasound.

### Results from the Imaging professionals

There were three key dominant themes from the Radiologists and Sonographers interviewed:

#### 1. Selective abortion of unwanted fetuses

All the participants in this category said that telling women the sex of the fetus at ultrasound may result into selective abortion of the fetus in case the mother did not want that particular sex. Most of the participants cited the experience they have had with women asking to know the fetal sex.

*“When I told the mother that she had a baby girl, she wished she had known earlier so as to abort the pregnancy since she said she had many girls and wanted a boy”, one sonographer said. In support of this, one Radiologist said, “Most of these mothers have pressure from society to carry a baby of a particular sex. When she learns that she is carrying something else, she opts for an abortion”*.

*“One disadvantage obstetric sonography has introduced is to accelerate the rate of abortions because women simply go for the scan without even a doctor's request simply to know whether they are carrying the right baby in terms of sex”, one sonographer expressed. Supporting the former, one radiologist added, “The idea of making money has made it worse. There are many ultrasound units and women simply go with money to know the fetal sex and they will be told.” “What is surprising is that it is in those very private clinics where health professionals carry out the abortions”, one sonographer conclusively said*.

#### 2. Psycho-social effects

This group of participants also cited psycho-social effects women may go through when told the fetal sex especially when they are told the opposite of what they want. These included stress, depression, social isolation, being branded an outcast and many more social effects. *“Most of these apply to those women who deliver only a particular type of gender e.g only boys or only girls”*, one Sonographer said. *“Most women who opt not to abort the fetus end up going through the pregnancy depressed emotionally carrying something they really do not want”*, one radiologist said.

However, it also becomes a conundrum when the mother delivers the opposite of what she was told during the scan. *“Some mothers do shopping in advance for a particular sex when they are told. There are many cases in which the mothers deliver the opposite of what they were told which becomes not only a social issue, but also a medico-legal one”, one participant said*.

#### 3. Need for policy

All the sonography professionals unanimously proposed the idea of establishing a nation-wide policy of not revealing fetal sex during obstetric scans unless it is necessary for medical reasons. *“We need the Ministry of Health to come up strongly on this issue and pass a policy barring any one from revealing fetal sex at ultrasound”*, one of them said. Another participant said, *“The policy should be operationalized to punish any culprits but not just remain on paper without being implemented”. “Bearing in mind that this involves monetary gains, the ministry should also sensitize the public about this contentious issue as well as the health professionals about the dangers of revealing fetal sex at ultrasound”*, another colleague said.

### Results from the recipients (women)

Opinions from this group of people revealed a different story as almost all the women wanted to know the fetal sex during the scanning before birth. There were two dominant themes from this group of participants:

#### 1. Early preparation

All women said that they would like to prepare themselves early enough before labour. On probing about the exact message in “preparation”, they said it is mainly shopping for the baby to come. *“The scan has helped us a lot since you can now shop things knowing the fetal gender, this helps not to waste money buying many unwanted items”*, one lady said. Another lady supported, *“Times have changed. We have financial limitations and we must only buy what is necessary, thank God the scan came into our lives.” “I have to send to nice things abroad and search around for a nice name for my baby. So I need to know early enough the sex for me to plan ahead of time”*, another lady said.

#### 2. Negative results

The second theme was put as “negative results”. Most of the women interviewed expressed some dissatisfaction with some scan results proclaiming that they have a baby girl only to deliver a baby boy and vice versa. *“During second pregnancy, I was told that I had a baby boy only to deliver a girl. Though I wanted to sue the health workers for stealing my baby, my husband discouraged from doing so saying that scans always tell lies”*, one lady recalled. Another lady said, *“I think there are many unqualified people doing this for business because after parting with a lot of money for the scan and being told that she had a baby boy, my sister delivered a girl. Good enough, she had bought unisex items for the start.”*

Despite all these observations, most of the women supported the idea of being told the fetal sex albeit by qualified people as it helps them prepare early as one lady summarily said: *“I have my own reservations for those results about the baby‘s sex from scans. However, it is a necessary evil and what I do is to have more than two scans from different places to compare.”* Asked about the financial implications of this, she concluded, *“But as long as it worth it, I do not mind.”*

## Discussion

Results from this study reveal two different lines of thought from two groups of participants. The imaging professionals think that revealing fetal sex should be discouraged while the recipients of the scan (women) still want to be told the fetal sex. From the perspective of the Radiologists and Sonographers, revealing fetal sex leads to selective abortions and psychological effects. In most Ugandan cultures, there exists the notion of gender bias where up to now males are slightly dominating and looked at as the future leaders. As a result of this, women will do whatever it takes to get a boy including aborting girls. Zechmeister [1] and Whynes [7] also reported that obstetric sonography has led to selective abortions of some babies in some cultures in preference to other babies of a desirable gender. This was also similar to what Kongnyuy et al [3] also advanced.

Such practice has also been accelerated by the ever increasing number of ultrasound facilities in both government owned and private health units. In most of these units, pregnant women just go for the scan just to know the fetal sex and as long as they pay, they are told. Like Mubuuke et al [23] reported, this trend raises numerous ethical dilemmas when mothers are told what they do not want to hear. The presence of numerous ultrasound training institutions has compounded this as well since many people are churned out to practice ultrasound. In many cases, this is looked at in terms of monetary gains and as long as the health professional is paid, the fetal sex will be revealed. Sometimes revealing fetal sex attracts more charge to the client as this is considered a “special” examination.

It should be strongly observed that revealing fetal sex during obstetric scans raises numerous psycho-social, ethical and legal dilemmas, in addition to how this knowledge of fetal sex affects a woman's emotions and relationship with her unborn child before and later on in life. It should be remembered that there are always false positives and false negatives with fetal sex which may have numerous implications like selective abortions of unwanted fetal genders. Williams et al [4] also warn about the ethical implications of telling pregnant women the fetal gender.

From the perspective of the women who receive the scan services, fetal sex should be revealed. It is understandable as to why women would want to know the fetal sex. In most cases it has been for them to prepare early in terms of shopping for the babies. In their study, Mubuuke et al [23] reported that many women actually go for the scan just to know the fetal sex. As a result of the desire to buy items early without spending a lot of money on unnecessary items, most women are likely to support the idea of telling them the fetal sex during the scans.

However, the women interviewed also raised the issue of false positives and false negatives. This was also raised by the Radiologists and Sonographers interviewed. It is true that many women are told the sex of the baby at ultrasound only to deliver the opposite and in some cases hospitals have been sued for this. This explains the need to regulate the practice of revealing fetal sex to the mothers by the concerned authorities like government ministries of health.

This study has revealed the implications of telling women fetal sex during obstetric scanning. Unfortunately this has become a monetary issue as well. If one health worker declines, the women go to another since there are numerous facilities doing obstetric sonography. In a developing society with a lot gender sensitivity issues, we propose a regulation been formulated and also implemented stopping diagnostic imaging professionals from revealing fetal sex to the mothers. Indeed fetal sex determination should not be part of the routine obstetric scan protocol unless it is justifiably requested for by a health worker strictly for medical reasons.

## Conclusion

Health care professionals including obstetricians, family physicians, general practitioners, midwives, and diagnostic imaging specialists should act to discourage the practice of sex selection. This can be done by strictly following a standard-of-care guideline for all pregnant women in which disclosure of fetal sex is not made (unless indicated for medical reasons).
